# Long-term farmyard organic residue amendments shape the resistance and resilience of soil microbial communities to simulated drying and heating-drying disturbances under a changing climate in the Senegalese groundnut basin

**DOI:** 10.3389/fmicb.2026.1888747

**Published:** 2026-09-16

**Authors:** Paul Ndiaga Ciss, Saïdou Nourou Sall, Laure Tall, Komi Assigbetse

**Affiliations:** 1 Initiative Prospective Agricole et Rurale (IPAR), Dakar, Sénégal; 2 Laboratoire des Sciences Biologiques, Agronomiques, Alimentaires et de Modélisation des Systèmes (LaBAAM), Unité de Formation et de Recherche en Sciences Agronomiques, Aquaculture et Technologies Alimentaire (UFR S2ATA), Department of Crop Productions and Agronomy, Gaston Berger University, Saint Louis, Senegal; 3 Laboratoire Mixte International Intensification Ecologique des Sols (LMI IESOL), Centre Institut Sénégalais de Recherches Agricoles-Institut de Recherche pour le Développement-Université Cheikh Anta Diop (ISRA-IRD-UCAD), Dakar, Sénégal; 4 Unité Mixte de Recherche Ecologie Fonctionnelle & Biogéochimie Des Sols & des Agroécosystèmes – Unité Mixte de Recherche Eco&Sols (Institut Agro Montpellier, Centre de coopération internationale en recherche agronomique pour le développement, Institut national de la recherche agronomique, Institut de recherche pour le développement), Montpellier, France

**Keywords:** arenosol, drying and heating-drying stress, farming practices, microbial communities, resilience, resistance, Senegal

## Abstract

**Introduction:**

Soil hosts a rich diversity of microorganisms essential to ecosystem functioning, but the droughts and high temperatures projected under future climate scenarios, particularly in Senegal, may significantly disrupt these processes. This study aimed to assess the effects of projected drying and combined heating-drying stress on the resistance and resilience of soil microbial communities, and to evaluate how these properties are shaped by contrasting farming practices, home-fields, which receive regular organic inputs, versus out-fields, which receive low or no organic inputs.

**Methods:**

Soils sampled from home-field and out-field plots were subjected to five cycles of drying and heating-drying disturbance under controlled conditions. The effects on carbon mineralization, β-glucosidase activity, and microbial biomass and abundance (PLFA profiles) were assessed.

**Results:**

Both drying and combined heating-drying significantly reduced microbial activity and biomass, regardless of farming practice, with soils exposed to drying alone less affected than those subjected to combined heating-drying stress. Long-term organic amendments in home-fields markedly enhanced soil microbial communities and improved resistance compared with out-fields receiving low organic inputs. Resilience patterns diverged sharply among microbial parameters: biomass and β-glucosidase activity showed low resilience, whereas carbon mineralization showed high resilience.

**Discussion:**

These results are consistent with reports that organic inputs enhance microbial resistance to drying-related stress, while the divergent recovery of carbon mineralization versus biomass and enzyme activity supports the view that functionally redundant processes remain buffered even when community structure does not fully recover. This underscores the value of long-term organic management for building drought resilience in Sahelian farming systems, and points to a need for field-scale, multi-cycle studies under recurrent climate extremes.

## 1 Introduction

Agriculture is the primary means of livelihood for populations in sub-Saharan Africa (Kinyili and Ndunda, 2022). This region is particularly exposed to the impacts of climate change, especially extreme events. Moreover, the frequency and intensity of floods, droughts, and heat waves are likely to increase as a result of global warming since several years (Hartmann et al., 2013). The IPCC (2022) projects heightened seasonal contrasts and decreased rainfall. The Senegalese groundnut basin has experienced these climatic disturbances, marked by a gradual decline in rainfall over the past two decades (Dione et al., 2008). This downward trend is characterized by recurrent drought episodes lasting between 10 and 15 days, with some extending up to 20 days (Salack et al., 2012). Additionally, pronounced temperature variability has been recorded in the region, with peak air temperatures reaching 43 °C (ANSD/SRSD Kaolack, 2015) and soil surface temperatures (0–10 cm) occasionally rising to 50 °C (AMMA-CATCH, 1990; Galle et al., 2018). The structure and functioning of microbial communities are strongly affected by climatic variability (Li G. et al., 2018). Temperature and soil moisture are considered the most influential environmental factors controlling soil microbial processes (Le Quéré et al., 2009). Previous studies have demonstrated that the effects of heat and moisture fluctuations on soil microorganisms (Bérard et al., 2012; Kumar et al., 2013), as well as changes in CO_2_ accumulation (Borken and Matzner, 2009), depend on both the frequency of heat–drying cycles and the intensity of the disturbance. De Nobili et al. (2006) reported that rising temperatures lead to a reduction in microbial biomass and alter carbon mineralization. Similarly, variations in soil moisture significantly affect microbial metabolism, abundance, and community composition (Kim et al., 2012).

However, soil microbial communities have developed various mechanisms that enable them to adapt to extreme weather events. Microbial community resistance and resilience indicate their capacity to sustain functions under disturbances (Orwin and Wardle, 2004). The microbial communities accelerate their respiration and metabolism to adapt to thermal disturbance (Chen et al., 2015). Re-wetting dry soils causes a rapid increase in CO_2_ emissions, intensified microbial activity and mineralization and therefore, plays an important role in the total carbon pool (Barnard et al., 2020).

Furthermore, there is a significant correlation between soil microbial activity and farming practices (Bandick and Dick, 1999; Deng and Tabatabai, 2000). These practices improve organic carbon content, physicochemical and enzymatic properties, and soil microbial metabolism. They contribute to the mitigation of climate change (Schlesinger and Amundson, 2019).

Numerous studies have investigated the effects of heat and water stress, particularly drying–rewetting cycles, on the microbial functioning of agricultural soils (Degens et al., 2001; Griffiths and Philippot, 2013; Pesaro et al., 2004; Steenwerth et al., 2005; Chaer et al., 2009). Most of this research has relied on field or laboratory experiments exposing temperate soils to varying levels of water stress. More recently, studies addressing the impacts of future climate change scenarios have also primarily focused on temperate regions (Schädler et al., 2019; Philipp et al., 2025). Soils in sub-Saharan Africa remain poorly studied (Ciss et al., 2023), particularly with regard to the frequency of heat–drying cycles and the intensity of stress expected from the combined effects of droughts and heat waves under future climate change. Studies simultaneously addressing these two effects in this area were not found. For the poor and fragile soils of this area, the addition of exogenous organic matter is a key lever for maintaining the soil’s organic status, which is essential for the productivity and sustainability of cultivated systems (Bationo et al., 2007). The application of organic amendments can improve the microbiological properties of cultivated soils (Sall et al., 2003; Mando et al., 2005; Freschet et al., 2008; Chotte et al., 2013).

Land use in Senegal’s groundnut basin is marked by two farming systems likely differ in several management and environmental factors, including cropping history, residue management, tillage, vegetation, spatial position and baseline soil properties (Manlay et al., 2004). Around each village, land is arranged in a series of concentric zones. Plots located near the concession, known as home-fields, receive substantially higher inputs than those situated farther away, referred to as out-fields. Distance from the houses is generally treated as the master variable. Everything else (tillage intensity, fallow vs. continuous cropping, tree cover, resulting soil fertility) follows from that single geographic fact, because proximity determines the labor cost of transporting manure, household waste, and effort to the plot. Farmers concentrate their limited fertilizing inputs and labor where the cost of doing so is lowest (around the houses). Manure and labor allocation were found as the proximate mechanisms linking this spatial position to soil and vegetation differences. Previous research by teams in our laboratory (Malou et al., 2020; Tounkara et al., 2020) showed, among several consequences, that home-fields consistently exhibit higher SOC than out-fields, a difference both studies attribute to differential manure and organic input allocation tied to proximity to the village. Home-field soils are regularly amended with organic matter–ranging from 4 to 20 Mg⋅ha^−1^ of dry matter from cattle and small ruminant manures–applied every 1 or 2 years. In contrast, out-field soils receive much lower levels of organic fertilization (≤1 Mg⋅ha^−1^⋅yr^−1^) and therefore remain poor in organic matter and nutrients (Malou et al., 2020; Tounkara et al., 2020).

The aim of this study was to assess the effects of projected drying and combined heating–drying stress on the resistance and resilience of soil microbial communities, and to evaluate how these two ecological properties are shaped by contrasting farming practices (home-field vs. out-field management). Resistance and resilience were assessed through changes in C mineralization, enzyme activity, microbial biomass and community structure (PLFA profiles). We hypothesized that microbial communities from home-field soils would exhibit greater resistance (i.e., smaller immediate declines in these variables following disturbance) and greater resilience (i.e., faster recovery of these variables toward pre-disturbance levels) to Drying and Heat–drying stress, compared to those from out-field soils.

## 2 Materials and methods

### 2.1 Sampling site

Soils were sampled from the home-fields and out-fields of Diohine, located in the groundnut basin at 14°29′51″N, 16°30′36″W. The study area is characterized by a Sudano–Sahelian climate, with a prolonged dry season extending from October to June and a short rainy season from July to September. Mean annual rainfall ranges between 400 and 600 mm, and the mean annual temperature is approximately 30 °C. The rainy season is marked by extreme heat, with maximum daily temperatures occasionally exceeding 43 °C (ANSD/SRSD Kaolack, 2015). Millet (*Pennisetum glaucum*) is the dominant crop, cultivated in both home-fields and out-fields, while other cereals and legumes are grown in rotation or intercropping systems. The woody vegetation is largely dominated by *Faidherbia albida* (Leroux et al., 2020).

### 2.2 Soil sampling strategy

Two (2) types of plots (home-fields and out-fields) were used which is representative of the two (2) cultural practices in groundnut basin. Plots located closer to the concession, considered home-fields, received larger amounts of inputs than the more distant plots, referred to as out-fields. The sampled plots ranged in area from 0.02 to 5.24 ha, with an average size of 0.5 ha (Malou et al., 2020). For each practice, 6 plots were randomly selected within the study area. In each plot, at least 5 soil samples were collected manually in a zig-zag pattern using an auger (0–10 cm depth) to form a composite sample per plot (approximately 6 kg of soil). The six replicate plots for each practice (home-field vs. out-field) were then pooled into a single composite sample (approximately 40 kg of soil) for the subsequent microcosm experiment.

Soils are classified as arenosols (IUSS/WRB, 2006) with generally low clay content, often less than 5%, mainly consisting of kaolinite (Malou et al., 2020).The soil samples were collected during the dry season and sieved to <2 mm and stored at room temperature pending processing. Physico-chemical characteristics of soils were performed at ISRA, CNRA laboratory of Bambey, Senegal (Ciss et al., 2023).

### 2.3 Stress and soil sampling strategy

The experiment was carried out under glasshouse-controlled conditions at the ISRA-UCAD-IRD research campus of Dakar (Senegal). Soil samples of home-field and out-field practices, were re-moistened to 70% of their water-holding capacity (WHC) and pre-incubated at 28 °C for two (2) weeks to stabilize microbial activity. After pre-incubation, 30 g of soil were collected and placed in 330 ml vials. For each farming practice, two (2) parameters were tested: temperature and drought. Thus, drying and heating-drying disturbances were applied to the soil samples based on CP4-Africa simulation model (AMMA-CATCH). This model reveals that in the future, the rainfall breaks will reach five, with respective durations of 11, 14, 13, 6, and 6 days. Under projected heat disturbance scenarios, the average soil temperature (0–10 cm depth) is expected to reach 50 °C.

For the stress application, the experimental unit of the microcosm experiment corresponds to the composite soil (*n* = 1 per practice), rather than the individual plot. The samples of each practice were divided into three treatments (each treatment repeated three times) : (i) Controls (C) were re-moistened to 70% of their WHC and incubated at 28 °C throughout the experiment. Humidity was readjusted every 3 days and maintained between 60% and 70% of the WHC, (ii) For drying (D) disturbance, samples were submitted to 5 wetting and drying (W/D) cycles of 11, 14, 13, 6, and 6 days, respectively, and incubated at 28 °C, (iii) for the heating–drying (HD) disturbance, samples underwent the same five wetting/drying (W/D) cycles used in the D treatment (durations of 11, 14, 13, 6, and 6 days, respectively), but were additionally incubated at 50 °C throughout each cycle (Figure 1). The bottles were opened during incubation. At the end of each cycle, soils were allowed to return to room temperature, and moisture was readjusted to 70% water-holding capacity (WHC) before the start of the next cycle. Microcosm temperature was monitored throughout incubation by reading the incubator’s LCD display at regular intervals, while soil moisture content was verified gravimetrically at the end of each cycle, to confirm agreement with the nominal target conditions. After completion of the W/D disturbance cycles, samples were subjected to a 26-day recovery incubation under control conditions (28 °C and 60%–70% WHC) (Figure 1). This microcosm experiment assessed specifically the cumulative and drastic effect of elevated temperature combined with drying (testing drastic, cumulative stress) on the resistance and resilience of microbial C mineralization, β-glucosidase activity and phospholipid fatty acids (PLFA).

**FIGURE 1:**
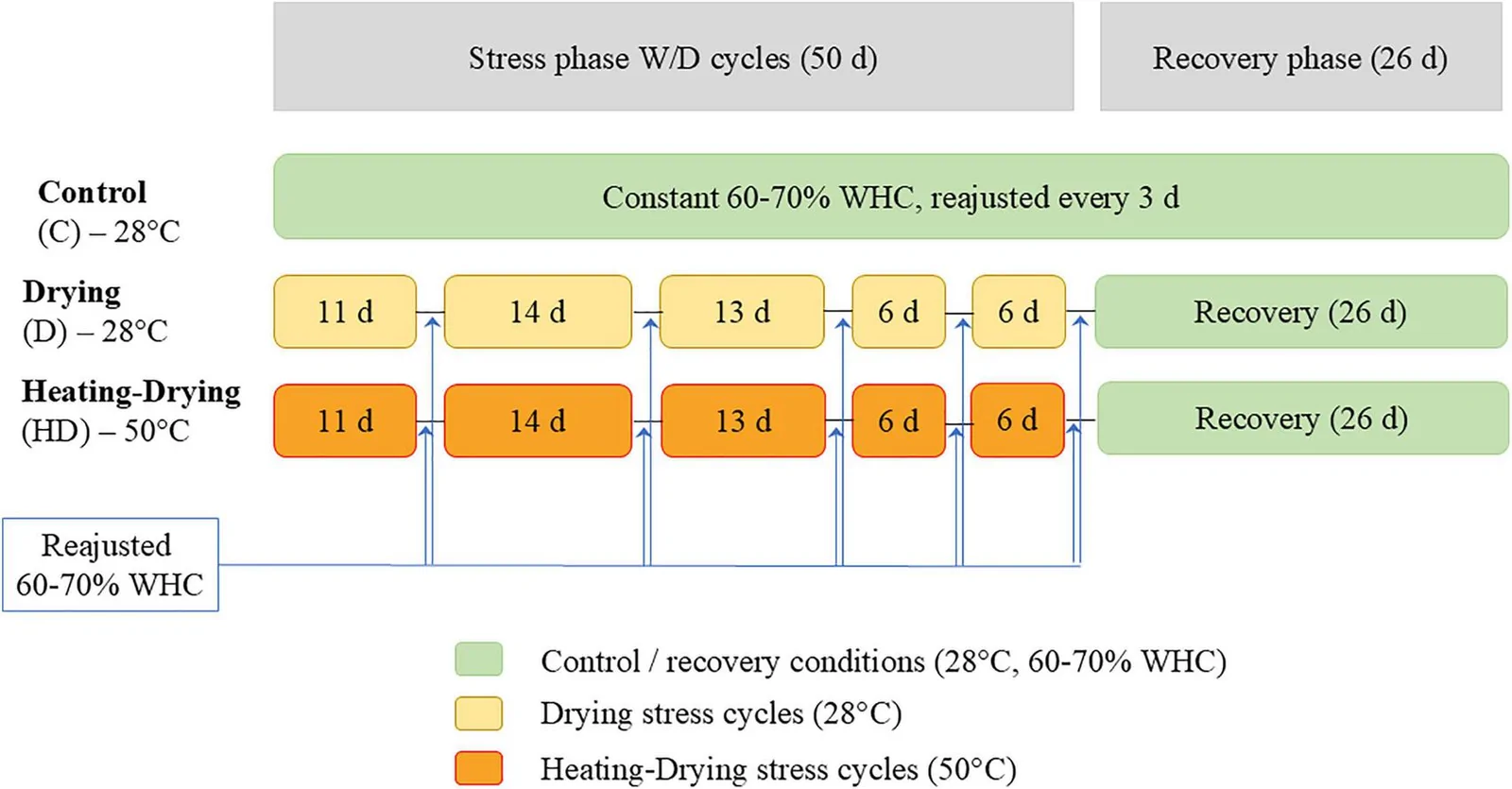
Schematic diagram (not to scale), illustrating the time course of drying disturbance and heating-drying disturbance (simulating drought and combining drought and heat wave events, respectively).

Measurements of C mineralization, β-glucosidase activity and phospholipid fatty acids (PLFA) were conducted at the Laboratoire Mixte International Intensification Ecologique des Sols (LMI IESOL) located on the IRD–ISRA Bel-Air campus in Dakar.

### 2.4 C mineralization

CO_2_ measurements quantify the metabolic activities of soil microorganisms (Nielsen and Winding, 2002). The concentration of C-CO_2_ in the ambient air of the vials was measured by direct injection using an Agilent 490 Micro GC instruments gas chromatograph (μ-CPG Agilent 490, 1109602). The vials opened during incubation were closed with waterproof rubber caps 2 h before each basal respiration measurement. During incubation, CO_2_ emissions from soil samples were measured daily (for the first 5 days) of incubation and then every 2 or 3 days until the end of the experiment. The total amounts of CO_2_ emitted (μg C-CO_2_ g^–1^ soil) were calculated after the 26-day recovery incubation for each treatment. C mineralization rates (μg C-CO_2_ g^–1^ soil.day^–1^) of a given soil sample were measured during the 26-day recovery incubation. The rates were also calculated immediately after stress.

### 2.5 β-glucosidase activity

β-glucosidase activity was measured using a modified colorimetric method (Hayano, 1973). The tests consist of 100 mg of fresh soil, 0.1 ml of citrate-phosphate buffer at pH 5.8, and 100 μl of para-nitrophenyl β D-glucopyranoside (concentration 5 mM; Sigma) mixed in tubes and then incubated with agitation at 37 °C for 2 h. The reaction is then stopped by alkalizing the medium by adding 3 ml of sodium carbonate (0.2 M Na_2_CO_3_). The soil suspension is centrifuged at 10,000 rpm for 5 min. The optical density of the sample is measured using a spectrophotometer (Thermo Scientific, GENESYS 20) at a 400 nm wave length. For each soil sample, the average concentration of para-nitrophenol (p-NP) from three replicates of the test is subtracted from that of the enzyme control and substrate control. The activity of β-glucosidase was expressed in μg p-NP g (soil)^–1^ h^–1^.

### 2.6 Phospholipid fatty acids (PLFA)

The modified high-throughput PLFA analysis method of Buyer and Sasser (2012) was used to measure microbial biomass and community composition in soil samples. Approximately 2 g of fresh soil from each sample was placed in 13 × 100 mm glass test tubes. After each series of 20 samples, an empty test tube was used as an analytical blank. The samples were dried in the SpeedVac. Lipids were separated by solid-phase extraction (SPE) using a 96-well SPE plate containing 50 mg of silica per well (Phenomenex 8E-S012-DGB). The PLFAs were collected with 0.5 ml of 5:5:1 methanol: chloroform: demineralized H_2_O in 1.5 ml microtubes placed on a multi-Tier plate. The analysis was performed using Agilent gas chromatography (Agilent Technologies, Wilmington, DE, USA) 6890N, with a system controlled by Agilent ChemStation software. Specific PLFA markers were used to determine the different microbial groups in the microcosms studied, using the standard fatty acid nomenclature described by Frostegård et al. (1993). Mor than 60 PLFAs were detected and identified in the various soil samples. Biomarkers for major microbial groups were determined by summing PLFAs as follows: bacteria gram− (15:1 w8c, 15:1 w6c, 15:1 w5c, 16:3 w6c, 16:1 w9c, 16:1 w7c, 16:1 w7c DMA, 17:1 w8c, 17:0 cy w7c, 16:0 2OH, 18:1 w9c, 18:1 w7c, 18:1 w6c, 18:1 w5c, 17:0 iso 3OH, 19:1 w8c, 19:0 cy w9c, 19:0 w7c, 20:1 w9c, 21:1 w9c, 21:1 w8c, 21:1 w4c, 21:1 w3c, 22:1 w8c, 22:1 w5c, 24:1 w9c), bacteria gram+ (11:0 anteiso, 14:0 iso, 15:1 iso w9c, 15:1 iso w6c, 15:0 iso, 15:0 anteiso, 16:0 iso, 16:0 anteiso, 17:1 iso w9c, 17:0 iso, 17:0 anteiso, 18:0 iso, 19:0 iso, 22:0 iso), actinobateria (16:0 10Me, 17:1 w7c 10Me, 17:0 10Me, 18:1 w7c 10Me, 18:0 10Me, 19:1 w7c 10Me, 20:0 10Me) and fungi (16:1 w5c, 18:2 w6c). Fatty acids with less than 0.5% of the total relative abundance were not included in the data set.

Total PLFA (PLFAtot) was used as an indicator of microbial biomass and was estimated by adding the masses of all fatty acids detected and was expressed in pico-moles of PLFA g^–1^ dry weight of soil (pmol g^–1^ soil) (Bossio and Scow, 1998).

### 2.7 Resistance and resilience indices

The indices of Orwin and Wardle (2004) were used to calculate the resistance (RS) and resilience (RL) of the microbial community. RS and RL of microbial communities in disturbed soil are better assessed by comparing their performance with that of undisturbed soil (Orwin and Wardle, 2004). Basal respiration and microbial biomass were used to quantify the resistance and resilience of the microbial community according to formulae 1 and 2:


$$\text{RS}\,\left(t\,0\right)=1-\frac{2\,l\,D\,0\,l}{\left(\mathrm{C0}+\mathrm{lD0l}\right)}$$ (1)

$$\text{RL}\,\left(t\,x\right)=\frac{2\,l\,D\,0\,\text{l}}{\left(\mathrm{lD0}\,\text{l}+\text{lD}\,x\,l\right)}-1$$ (2)

Where *D0* is the difference between the control (*C0*) and the stressed (*P0*) for each cycle. The RS was calculated at the end of the first W/D cycle and at the end of the last W/D cycle (5th W/D cycle). *Dx* is the difference between the control (*Cx*) and the disturbed soil (Px) at *x* = 26 days (end of incubation). The RS and RL indices range from −1 to +1. A value of 1 indicates either total resistance (RS; no effect of the disturbance) or complete resilience (RL; full recovery after disturbance), whereas values close to 0 reflect low resistance or incomplete recovery. Indices approaching 1 therefore denote high resistance or rapid recovery. When the value of P0 exceeds twice the control value (2C0), the index becomes negative. Negative values indicate a decline rather than a recovery. This situation may arise when the disturbance initially reduces the measured response variable but simultaneously increases substrate availability, resulting in a subsequent strong stimulation of the response.

### 2.8 Statical analyses

Statistical analyses were performed using XLSTAT-Pro software (version 6.1.9, Addinsoft, 2002). Prior to analysis, data were tested for homogeneity of variances using Bartlett’s test and for normality of residuals using the Shapiro–Wilk test. A one-way analysis of variance (ANOVA) was then performed. Multiple comparisons were carried out between treatments within the two distinct farming practices (home-field and out-field), as well as between the same treatment across both practices. However, the experimental unit of the microcosm experiment corresponds to the composite soil (*n* = 1 per practice), rather than the individual plot. Microcosm replicates prepared from each composite soil constitute technical replicates, allowing assessment of variability associated with experimental handling, but do not capture the spatial variability among the original plots.

Mean separation was performed using Fisher’s least significant difference (LSD) test at the 5% probability level. For all analyses, statistical significance was set at *p* < 0.05.

## 3 Results

### 3.1 Effect of stress on soil C mineralization

Cumulative CO_2_–C production (μg CO_2_–C g^−1^ soil) was quantified after 26 days of recovery. In both home-fields and out-fields, control (C) soils exhibited significantly higher cumulative CO_2_–C respiration than soils subjected to drying (D) and heating-drying (HD) stress (Figure 2). In addition, cumulative CO_2_–C respiration under the D treatment was significantly greater than under the HD treatment. Overall, home-field soils consistently showed higher cumulative CO_2_–C respiration than out-field soils across all treatments.

**FIGURE 2:**
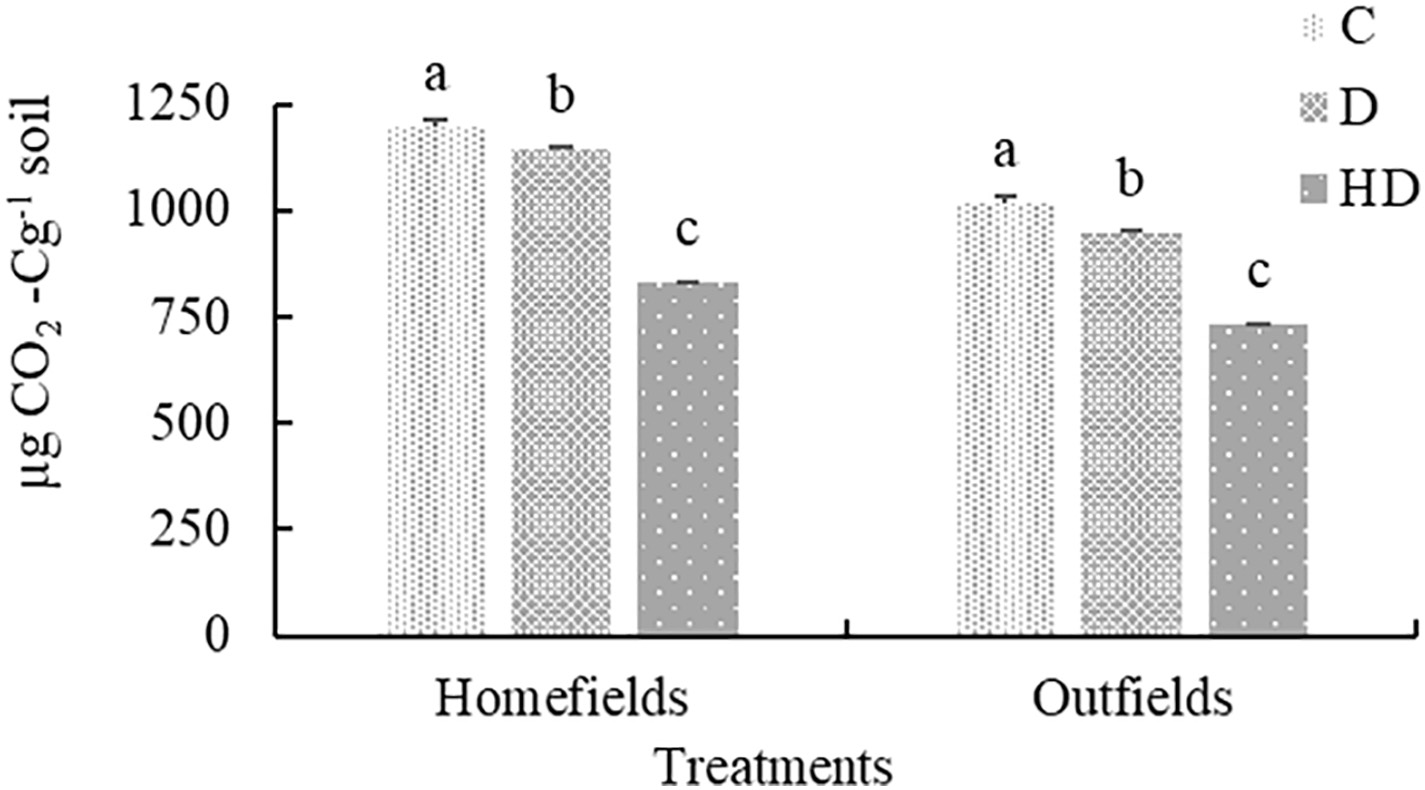
Cumulative CO_2_-C mineralized in soils from the home-fields and out-fields. Within each cultivation practice, different letters indicate significant differences between the three treatments, (*p* < 0.05) according to Fisher’s test (LSD). C, control (no stress); D, drying disturbance; HD, heating-drying disturbance.

After the 1st disturbance cycle, significant decrease in CO_2_-C fluxes was observed for HD treatment compared to the C soil both in home-field and out-fields soils (Table 1). In contract, after 5 cycles of perturbation, CO_2_-C fluxes increased in D treatment (18.88 CO_2_-C g^–1^ soil h^–1^) and HD treatment (21.09 CO_2_-C g^–1^ soil h^–1^) compared to C (16.73 CO_2_-C g^–1^ soil h^–1^) in home-field soils. While, in out-field soils, the CO_2_-C fluxes increased in D treatment (15.51 CO_2_-C g^–1^ soil h^–1^) and decrease in HD treatment (12.29 CO_2_-C g^–1^ soil h^–1^) compared to the C soil (13.66 CO_2_-C g^–1^ soil h^–1^). For both home-field and out-field soil, D treatment exhibited higher CO_2_ fluxes than HD treatment at the end of the incubation (Table 1). Throughout the incubation, CO_2_–C fluxes from home-field soils (C, D and HD) were consistently higher than those from out-fields soils, particularly after 1st and 5th disturbance cycles. However, at the end of incubation, CO_2_–C fluxes from home-field soils and out-field soils were not different.

**TABLE 1:** Soil C mineralization rates, β -glucosidase and Microbial biomass in home-field and out-field plots at different incubation stages: the first cycle, the fifth cycle, and at 26 days (end of incubation).

	Flux of CO_2_-C				β -glucosidase			Microbial biomass		
	μ g CO_2_-C g^–1^ soil h^–1^				μ g p-Np g^–1^ h^–1^			pmol 10^3^ g^–1^ soil		
Farming Practices	Stress treatment	1^st^ cycle	5^th^ cycle	26 days	1^st^ cycle	5^th^ cycle	26 days	1^st^ cycle	5^th^ cycle	26 days
Home-field	C	32.9 a	16.73 c	10.03 ab	22.33 a	24.24 a	20.64 a	22.612 a	15.475 b	14.071 b
	D	32.87 a	18.88 b	10.25 a	26.34 a	24.75 a	19.31 a	23.747 a	22.260 a	20.850 a
	HD	23.86 b	21.09 a	9.80 a	19.90 a	16.07 b	16.63 a	20.250 a	18.696 ab	15.754 ab
Out-field	C	20.04 a	13.66 b	10.04 ab	6.94 a	5.07 a	5.8 a	14.603 a	14.071 a	15.924 a
	D	17.99 a	15.51 a	10.24 a	9.13 a	7.43 a	5.19 a	11.565 ab	12.033 ab	12.456 a
	HD	16.67 b	12.29 c	9.94 b	7.07 a	6.85 a	3.54 a	8.351 b	6.367 b	10.147 b

In each cultivation practice (home-fields or out-fields), the different letters indicate significant differences between C, D and HD (*p* < 0.05) with C, control; D, drying disturbance; HD, heating-drying disturbance.

The RS indices under the drying (D) treatment were higher than those under heating-drying (HD) treatment, with a significant difference observed at the end of the 1st cycle for both home-fields and out-fields, and at the end of 5th cycle for the home-field (Table 2). Accordingly, C mineralization showed lower resistance to HD disturbance than to D disturbance in field practices. Resilience indices were high in home-fields under both D and HD disturbances (0.89 and 0.95, respectively) and in out-fields under HD disturbance (0.96) (Table 2). In contract, the D treatment in out-field showed low resilience (0.36). Overall, home-fields soils displayed high resilience under both disturbances, whereas in out-fields soils, high resilience was observed only under HD disturbance after 26 days of recovery.

**TABLE 2:** Resistance (RS) and resilience (RL) indices of C mineralization, β -glucosidase and microbial biomass to drying and heating-drying disturbance of home-field and out-field soils.

		C mineralisation			β -glucosidase			Microbial biomass		
Treatment	Stress	RS		RL	RS		RL	RS		RL
Farming Practices	Stress treatment	1^st^ cycle	5^th^ cycle	26 days	1^st^ cycle	5th cycle	26 days	1^st^ cycle	5^th^ cycle	26 days
Home fields	D	0.63 a	0.52 a	0.52 a	0.57 a	0.89 a	0.11 a	0.87 a	0.46 a	−0.68 a
	HD	0.31 b	0.33 b	0.33 b	0.75 a	0.50 a	−0.08 a	0.85 a	0.51 a	−0.41 a
Out fields	D	0.84 a	0.60 a	0.60 a	0.39 a	0.39 a	0.03 a	0.38 a	0.66 a	0.39 a
	HD	0.35 b	0.48 a	0.48 a	0.29 a	0.33 a	0.50 a	0.48 a	0.42 b	−0.02 a
Treatment^a^		*	ns	***	ns	ns	ns	***	ns	ns
Stress		***	*	***	ns	ns	ns	ns	ns	ns
Treatment*stress		ns	ns	***	ns	ns	ns	ns	*	ns

Within each cultivation practice (home-field or out-field), different letters indicate significant differences between D and HD (*p* < 0.05). D, drying disturbance; HD, heating-drying disturbance. Indexes RS (Resistance) and RL (Resilience) were calculated according to Orwin and Wardle (2004).

^a^*F*-values are significant at 0.05

(*) and 0.001

(***) probability levels. *ns* is not significant.

### 3.2 Effect of future stress on β-glucosidase activity

β-glucosidase activity was measured at the end of the 1st and 5th cycles and at the end of the incubation period (26 days). At the end of the 5th cycle, β-glucosidase activity in the control (C) and drying (D) treatments of the home-field soils was significantly higher than in the heating-drying (HD) treatment (Table 1). No significant differences were observed among the others treatments in either home or out-fields. Overall, enzyme activity was consistently higher in home-field soils than in out-field soils across the different measurement times (Table 1).

No significant differences were found for RS indices of β-glucosidase activity between drying (D) and heating–drying (HD) treatments in home-field and out-field soils at the 1st and 5th cycle, although RS indices were the lowest in out-field soils (Table 2). The RL indices for β-glucosidase indicated incomplete recovery of enzymatic activity in home-field out-field soils. Moreover, the RL index of the HD treatment in out-field soils (0.50) was significantly higher than that observed in the corresponding home field treatment (- 0.08) (Table 2).

### 3.3 Effect of stress on the microbial biomass and communities

#### 3.3.1 Microbial biomass

In home-field soils, microbial biomass under the drying (D) treatment was significantly higher than under the control (C) after the 5th cycle and at the end of incubation (Table 1). In contrast, the biomass under the heating-drying (HD) treatment did not differ significantly from that under the C and D treatments at any measurement cycle. In out-field soils, microbial biomass in the control (C) treatments was significantly higher than in the HD treatment across all measurements cycle (Table 1). Furthermore, microbial biomass in the D and HD treatments of home-field soils was higher than in the corresponding out-field treatment at the end of the 1st and 5th cycles and at the end of incubation.

After the 1st cycle, RS indices for the D and HD treatments did not differ significantly in either field type (Table 2). For both disturbance, RS values in home-field soils were higher than those in out-field soils at the end of the first cycle (Table 2). At the end of the 5th cycle, however, the RS indice of D treatment in out-field soils (0.66) was significantly higher than that of the HD treatment (0.42). Overall, the results indicate an approximately 50% reduction in microbial biomass after this cycle.

The RL indices for the D and HD treatments in home-field soils (−0.68 and −0.41, respectively) indicated a lack of resilience. In contrast, RL indices for out-field soils treatments (0.39 and −0.02 for D and HD, respectively) reflected only partial recovery of microbial biomass after 26 days of recovery (Table 2).

#### 3.3.2 Abundance of microbial communities

At the end of the 1st cycle, the abundance of Actinobacteria in control (C) and drying (D) treatments of home-field soils was significantly higher than in the HD treatment. In contrast, fungal abundance in home-field soils was significantly higher under the D and HD than the C treatment (Table 3). No significant difference among treatments were observed in out-field soils for any microbial group (Table 3).

**TABLE 3:** Abundance of microbial communities in the home-field and the out-field soils measured after the 1st cycle and the end of incubation.

Farming Practices	Stress treatment	Gram+ bacteria	Gram− bacteria	Actinobacteria	Fungi
		(pmol 10^3^ g^–1^ soil)			
1st cycle					
Home-field	C	6.682 ab	6.377 a	4.256 a	0.692 b
	D	7.209 a	6.254 a	4.072 a	0.746 a
	HD	5.797 b	6.292 a	2.754 b	0.737 a
Out-field	C	4.291 a	3.704 a	3016.81 a	0.441 a
	D	3.478 a	3.414 a	1607.73 a	0.333 a
	HD	3.043 a	1.488 a	1384.08 a	0.339 a
End of incubation					
Home-field	C	4.632 a	2.862 a	2.358 a	0.397 a
	D	6.235 a	5.724 a	3.369 a	0.625 a
	HD	4.653 a	4.339 a	2.621 a	0.509 a
Out-field	C	4.621 a	4.096 a	3.637 a	0.229 a
	D	3.919 a	3.202 ab	2.447 b	0.219 a
	HD	2.562 b	2.200 b	0.765 c	0.138 b

Microbial groups (Gram+ bacteria, Gram− bacteria, Actinobacteria, and Fungi) were obtained by summing their corresponding biomarkers (Frostegård et al., 1993). Within each cultivation practice (home-field or out-field), different letters indicate significant differences between C, D, and HD treatments (*p* < 0.05). C, control; D, drying disturbance; HD, heating-drying disturbance.

At the end of incubation (26 days), no significant treatments differences were detected in home-field soils for any microbial community parameter (Table 3). In the out-field soils, however, Gram+ bacteria, Gram− bacteria, Actinobacteria and fungi in the C treatment were significantly higher than in the HD treatment (Table 3). When comparing cultivation practices, Gram+ bacteria and fungi in the D and HD treatments were more abundant in home-field soils than in the corresponding out-field soils (Table 3), while Gram− bacteria and Actinobacteria were less abundant.

#### 3.3.3 Changes in microbial communities at the end of the disturbances

The percentages of change in microbial community abundance were calculated by the changes induced by drying (D) and heating-drying (HD) disturbance compared to the control (Figure 3).

**FIGURE 3:**
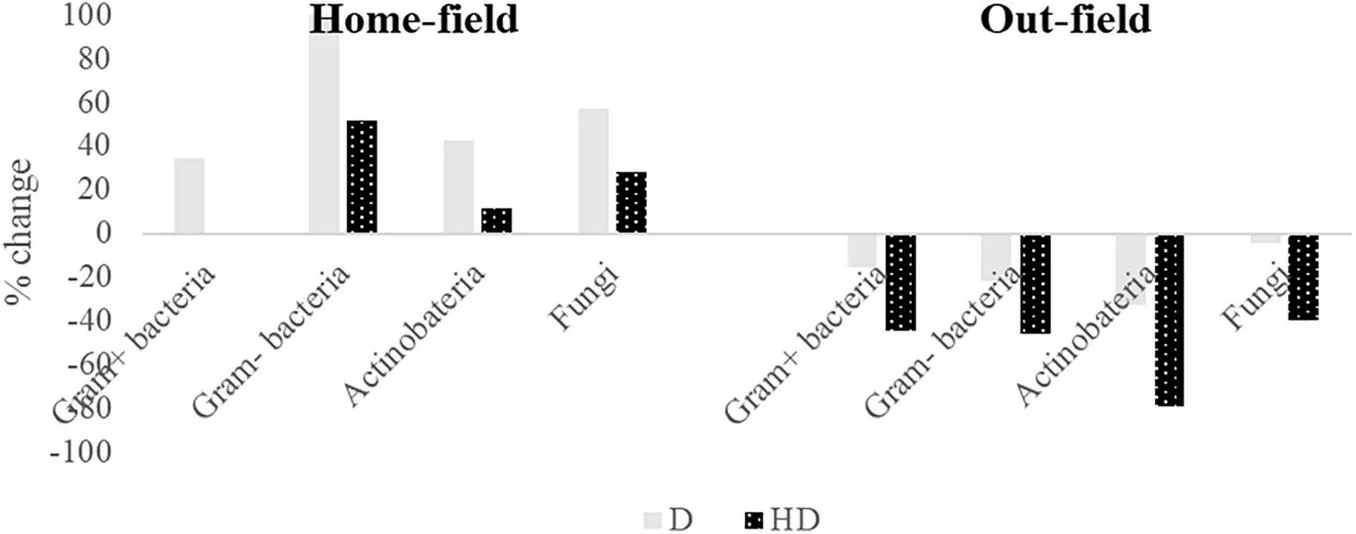
% changes in microbial communities between stressed treatments and the control in the home-field and the out-field soils at the end of incubation. D, drying disturbance; HD, heating-drying disturbance.

In home-field soils, Gram+, Gram−, Actinobacteria and Fungi increased in the D treatment (by +34.6%, +99.9%, +42.9% and +57.4%, respectively) and in the HD (by +0.5%, +51.6%, +11.2% and +28.3%, respectively). In these soils, microbial communities increased significantly under D treatment compared to HD, particularly Gram−.

For out-field soils, Gram+, Gram−, Actinobacteria and fungi decreased in the D treatment (−15.2%, −21.8%, −32.7% and −4.2%, respectively) and in the HD (−44.6%, −46.3%, −78.9% and −39.8%) (Figure 3). In these soils, microbial communities have declined significantly due to cumulative HD treatment. Actinobacteria were more affected.

## 4 Discussion

For the entire period of incubation, cumulative CO_2_-C mineralized in the home-field soils is higher than in the out-field soils. This result might be explained by the carbon content of these soils. Tounkara et al. (2020) estimated the organic inputs from home-field soils in the range of 4–20 Mg ha^−1^ of dry matter, while out-field soils received an annual amount of organic matter less than 1 Mg ha^−1^. Long-term application of organic wastes significantly affects the total carbon content in these soils. Chotte et al. (2013) found significant C mineralization in soils amended with organic residues compared to unamended soils. This availability of organic C in home-field soils also explains the higher microbial biomass in these soils compared to out-field soils. Several authors showed that C mineralization of a given soil is positively correlated with its carbon content, microbial biomass and activity (Sarmiento and Bottner, 2002; Xie et al., 2022; Guo et al., 2023; Shu et al., 2023).

During the whole incubation periods, soils were differently affected by perturbation (drying and heat-drying). The total amount of CO_2_-C mineralized in the control soils was higher than in stressed soils and whatever the farming practice (home-fields or out-fields). Several studies have shown similar results (Canarini and Dijkstra, 2015; Li J. T. et al., 2018; Muhr et al., 2010). Yin et al. (2019) explains this by a reduction in the rate of decomposition of organic matter due to disturbances. However, in our study, the rate of decomposition varied according to the cycle. For the first cycle, the combined effect of HD soils, significantly decreased the CO_2_-C mineralized compared to control soil (C). Whereas, D did not affect C-mineralization at the 1st cycle of disturbance. The combined stress showed a stronger effect than only drying (Bérard et al., 2011; Fikri et al., 2021). These authors suggested that this is probably due to stress-induced cell desiccation, cell lysis, and the thermal denaturation of enzymes. D stress had less impact on microbial activity than HD stress. The resistance indices confirmed the hypothesis and showed RS higher in the soil stress by D compared to soil with combined HD. Some authors have shown that soil microorganisms frequently exposed to drying–rewetting cycles are less affected by additional D stress (Fierer et al., 2003; Bouskill et al., 2013). The combined drying and heating could accelerate the decrease in soil moisture. There is a positive correlation between moisture and microbial activity in soil. Soil moisture is a crucial environmental factor influencing microbial metabolism in soil (Blazewicz et al., 2014; Li J. T. et al., 2018). Resistance indices calculated showed that home-field soils were less affected by disturbance indicative that organic inputs mitigate the inhibitory effect of disturbance on soil microbial biomass and activities. Wada and Toyota (2007) showed that soil amended with manure for many years was more resistant than unamended soil. However, it had been showed that these effects depended to the disturbance type, intensity and duration (Schimel et al., 2007; Bérard et al., 2011). Long-term soil disturbance induced by D and HD appears to increase C substrate availability for soil microorganisms (Borken and Matzner, 2009), potentially stimulating microbial metabolism and decomposition, reflecting a pulse of C mineralization following the 5th cycle. This response was mainly observed in soils rich in organic matter, such as home-field soils. In contrast, although D enhanced C mineralization in out-field soils, the long-term application of combined HD still resulted in reduced C mineralization in these soils, which are characterized by low organic carbon content. In these low-C soils, prolonged disturbance likely led to substrate limitation and greater microbial stress, thereby constraining microbial activity and C mineralization despite rewetting. In our study, the measurement of dissolved organic carbon (DOC) in soils after disturbance could help explain this discrepancy, as DOC represents the readily available carbon pool for microbial metabolism (Bérard et al., 2011). These results were supported by the increase in microbial biomass observed in stressed soils from home-fields, likely due to greater C substrate availability for microbial metabolism, whereas biomass decreased in out-fields compared with unstressed soils after the 5th cycle and at the end of the incubation period. Several authors have reported a significant decline in microbial biomass following heat stress (Allison and Martiny, 2008; Riah-Anglet et al., 2015). This reduction is primarily attributed to microbial cell death and degradation driven by increased energy demands (Joergensen et al., 1990). In outfields, these energy requirements are not met due to low organic matter inputs, resulting in limited substrate availability for microbial communities. The determination of the metabolic quotient (qCO_2_) could help explain this pattern, as qCO_2_ reflects the availability of carbon substrates and their allocation between microbial maintenance energy demand and biosynthesis under stress conditions (Anderson, 2003). Unfortunately, PLFA-based estimates of microbial biomass do not enable precise calculation of qCO_2_, as they are highly sensitive to variations in microbial community composition and physiological state (Frostegård et al., 2011).

When comparing home-field and out-field soils at the end of the incubation, both D and HD stresses irreversibly altered microbial biomass and β-glucosidase activity, indicating low resilience of these microbial properties. In contrast, C mineralization showed high resilience. This confirms that microbial communities surviving of different perturbations display metabolic activity distinct from that of communities present prior to disturbance, as reported in an earlier study (Chotte et al., 2013). And, no significant effects of organic inputs on the resilience of soil microbial biomass and β-glucosidase activity were observed in this study.

It is noteworthy that, in home-field soils, all microbial groups (Gram+ bacteria, Gram− bacteria, Actinobacteria, and fungi) increased by the end of the incubation, regardless of the perturbation, with the highest increases observed for Gram-negative bacteria and fungi under the D treatment. In contrast, in out-field soils, all microbial groups declined regardless of the perturbation, with the greatest reduction observed for Actinobacteria under the HD treatment. The perturbations appear to have altered differently microbial community composition depending on soil status (home vs. out-field). We hypothesize that this reflects both the elimination of disturbance-sensitive species and the selection of tolerant ones, as well as a subsequent proliferation of fast-growing populations once favorable soil conditions were restored (Bérard et al., 2012). Fast-growing microorganisms, often described as r-strategists, are adapted to periods of rapid growth driven by the availability of easily accessible substrates (Ho et al., 2017). We suggest this may explain the patterns observed in home-field soils following perturbation. In nutrient-poor soils, these organisms would either die or enter dormancy, as they are unable to efficiently utilize soil organic matter, whereas microorganisms that rely on soil organic matter, classified as K-strategists, would be expected to persist under such conditions (Ho et al., 2017; De Vries and Shade, 2013). Their slow growth rate reflects a distinct pattern of energy allocation (Lipson, 2015), with greater investment in extracellular enzyme production and defense against predation rather than in biomass production. Disturbance tends to reduce these populations (Grall and Coïc, 2005).

A limitation of this study lies in the pooling of the six replicate plots into a single composite soil per practice, which restricts the statistical inference that can be drawn. The results obtained reflect the response of two composite soils with contrasting management histories (home-field vs. out-field), rather than a statistically generalizable comparison across all plots of each practice within the study area. The differences observed between the two composite soils should therefore be interpreted as trends associated with distinct management histories, rather than as statistically significant effects at the landscape scale. Future studies incorporating non-pooled plots as independent replicates would help confirm the robustness and generalizability of these findings.

## 5 Conclusion

This study assessed whether the two farming systems (home-field and out-field) in Senegal’s groundnut basin can withstand the drying and combined drying–heat stresses projected under climate change.

Prolonged disturbance, whether drying alone (D) or combined heat–drying (HD) significantly reduced microbial activity and biomass in cultivated soils regardless of the farming system, with heat–drying stress causing consistently greater impacts than drying alone. Long-term organic amendments in home-fields soils, however, markedly enhanced soil microbial communities and conferred greater resistance to these disturbance than in out-field soils, which lacked organic inputs.

By the end of the recovery period, resilience indices diverged sharply among microbial parameters: biomass and β-glucosidase activity showed low resilience, whereas C mineralization showed high resilience. This divergence suggests that the microbial communities surviving disturbance were functionally and metabolically distinct from those present before disturbance, rather than simply a recovering subset of the original community. Notably, long-term organic inputs had no detectable effect on the resilience of soil microbial biomass or enzyme activity, likely, because recovery from the preceding disturbance remained incomplete within our experimental window. This points to a duration limitation rather than an absence of effect: a longer recovery period beyond the 26 days used here may be needed to capture the full resilience trajectory of these communities, particularly for slower-recovering parameters such as biomass and enzyme activity.

Several methodological constraints should also be considered when interpreting these results. The microcosm design likely introduced confinement effects and excluded plant–microbe interactions and active carbon inputs from root exudation or litter, both of which may have limited the recovery capacity of microbial biomass and functional redundancy relative to field conditions. Future work incorporating shotgun metagenomic sequencing would allow a more comprehensive characterization of taxonomic and functional diversity shifts underlying the activity and biomass responses observed here.

## Data Availability

The datasets presented in this study can be found in online repositories. The names of the repository/repositories and accession number(s) can be found below: The raw data supporting this study will be made available by the authors upon request to interested researchers.
